# Preparation and Evaluation of a Novel Class of Amphiphilic Amines as Antitumor Agents and Nanocarriers for Bioactive Molecules

**DOI:** 10.1007/s11095-016-1999-9

**Published:** 2016-07-25

**Authors:** Isabella Orienti, Mirella Falconi, Gabriella Teti, Mark A. Currier, Jiang Wang, Mitch Phelps, Timothy P. Cripe

**Affiliations:** 1 Department of Pharmacy and Biotechnology, University of Bologna, Via S. Donato 19/2, Bologna, Italy; 2 Department for Biomedical and Neuromotor Sciences (DIBINEM), University of Bologna, via Irnerio 48, Bologna, Italy; 3 Center for Childhood Cancer and Blood Diseases, Nationwide Children’s Hospital, Columbus, Ohio USA; 4 College of Pharmacy and Division of Pharmaceutics, The Ohio State University Comprehensive Cancer Center, Columbus, Ohio 43210 USA; 5 Division of Hematology/Oncology/Blood and Marrow Transplant, Nationwide Children’s Hospital, 700 Children’s Dr, Columbus, Ohio 43205 USA

**Keywords:** antitumor activity, encapsulation of bioactive molecules in micelles, formation of micelles in water, novel amphiphilic amines, pharmacokinetics and biodistribution

## Abstract

**Purpose:**

We describe a novel class of antitumor amphiphilic amines (RCn) based on a tricyclic amine hydrophilic head and a hydrophobic linear alkyl tail of variable length.

**Methods:**

We tested the lead compound, RC16, for cytotoxicity and mechanism of cell death in several cancer cell lines, anti tumor efficacy in mouse tumor models, and ability to encapsulate chemotherapy drugs.

**Results:**

These compounds displayed strong cytotoxic activity against cell lines derived from both pediatric and adult cancers. The IC50 of the lead compound, RC16, for normal cells including human keratinocytes, human fibroblasts and human umbilical vein endothelial cells was tenfold higher than for tumor cells. RC16 exhibited significant antitumor effects *in vivo* using several human xenografts and a metastatic model of murine neuroblastoma by both intravenous and oral administration routes. The amphiphilic character of RC16 triggered a spontaneous molecular self-assembling in water with formation of micelles allowing complexation of Doxorubicin, Etoposide and Paclitaxel. These micelles significantly improved the *in vitro* antitumor activity of these drugs as the enhancement of their aqueous solubility also improved their biologic availability.

**Conclusions:**

RC16 and related amphiphilic amines may be useful as a novel cancer treatment.

**Electronic supplementary material:**

The online version of this article (doi:10.1007/s11095-016-1999-9) contains supplementary material, which is available to authorized users.

## Introduction

Newer, targeted antitumor drugs currently in clinical use have improved the outcome of many cancers by exploiting different mechanisms of action to overcome resistance to chemotherapy (1,2). However drug toxicity is often dose limiting, reducing the chance to kill all tumor cells before they develop multidrug resistance. Pediatric tumors in particular represent a serious challenge to chemotherapy as they often grow fast and rapidly develop resistance (3). Also, many types of adult tumors including breast, colon and lung carcinoma frequently fail to respond to therapeutic treatments due to drug resistance (4,5).

Among antitumor agents in development, the lipophilic amines (6) appear particularly interesting. Their mechanism of action is mainly located in mitochondria and lysosomes, unlike inhibitors of DNA, RNA or RTKs that often induce resistance through the expression of P-glycoprotein, multidrug-resistance associated proteins or activation of alternative RTK pathways (7). The alterations in mitochondria and lysosomes that occur in cancer cells (8–18) make the anticancer effect of the lipophilic amines quite specific. A major drawback to their clinical use, however, is their high hydrophobicity, which restrains their bioavailability and consequently their therapeutic efficacy.

We hypothesized that amphiphilic amines (RCn) whose molecular structure comprises a tricyclic amine hydrophilic head and a hydrophobic linear alkyl tail of variable length would be harmful to cancer cells and serve as drug-delivery vehicles. Our primary objective was to determine the antitumor activity of these novel agents alone in both pediatric and adult cell lines and animal tumor models. Mice were used because of the availability of human tumor xenograft models. Our secondary objective was to determine the ability of these agents to be complexed with other anticancer agents such as chemotherapy. Thus, RC_16_ was also evaluated as a nanocarrier for bioactive molecules as, due to its amphiphilic character, it spontaneously self-assembles in water into micelles able to complex hydrophobic drugs.

## Materials and Methods

### Materials

DBC was purchased from Sigma-Aldrich. All the other reagents and solvents for the synthesis of the amphiphilic amines were purchased from Fluka.

### Cell Lines

Human cancer cell lines 143.98.2, A673, A549, CHLA20, S462TY and Ramos have been previously described (19–22). Normal primary HUVECs, Human Foreskin Keratinocytes (differentiated) were also previously described (22,23). Alveolar rhadomyosarcoma cell line Rh41 was obtained from the Pediatric Preclinical Testing Program (Peter Houghton, Nationwide Children’s Hospital) and maintained in RPMI supplemented with 10% fetal bovine serum (FBS), l-glutamine, and 100 IU/ml penicillin and 100 μg/ml streptomycin. Murine neuroblastoma cell line Neuro2A, normal gingival fibroblasts (24), and the human cancer cell lines SK-N-AS (neuroblastoma), MDA-MB-231 (breast) and WIDr (colon adenocarcinoma) were purchased from ATCC (Manassas, VA). All cells purchased from the ATCC were maintained according to supplier instructions.

### Animal Studies

All animal studies were approved by the IACUC at Cincinnati Children’s Hospital Medical Center (prior to the lab’s relocation to Nationwide Children’s Hospital). Athymic nude (nu/nu) and A/J female mice, 4–6 weeks old, weighing 20–25 g were purchased from Harlan Sprague Dawley (Indianapolis, IN). Athymic nude mice were used to determine RC_16_ maximum tolerated dose, pharmacokinetics, drug biodistribution and efficacy in xenograft tumor models. A/J mice were used to determine efficacy in an immunocompetent tumor model. Animals were housed 4 mice/cage in the Cincinnati Children’s Hospital Medical Center AAALAC accredited facility. Mice were kept in specific pathogen free rooms, which maintain constant temperature, humidity, and light/dark cycle. Animals were observed daily by the animal care staff including weekends. A full-time veterinarian was available to ensure adequate veterinary care. Animals were provided water and chow *ad libitum*. Cages and bedding were changed once/week and all cages were maintained in racks with independent sterile water and hepa-filtered airflow.

### Determination of the Maximum Tolerated Dose in Mice

To determine the MTD, a standard assay dose escalation study was performed. Non-tumor bearing, female, athymic nude mice were injected once with RC_16_ by the tail vein starting with 0.5 mg/kg. The doses were escalated by 0.25 mg/kg until MTD was achieved. Following convention, each dose was tested on a cohort of 5 mice in individual independent experiments. MTD was defined as the highest dose that could be given resulting in no drug-related moribund state or death or body weight loss ≥ 20% in the first 7 days. Other signs of toxicity such as unusual mouse behavior, lack of movement and poor posture were also monitored. Mice were weighed and observed 3 times per week for any signs of toxicity. Mice that demonstrated signs of toxicity were humanely euthanized by CO_2_ asphyxiation.

### Pilot Pharmacokinetic Study in Mice

To establish a standard assay for RC_16_ detection and the half-life of RC_16_ in murine blood, a basic pilot pharmacokinetic study was performed using 1 animal per timepoint. Non-tumor bearing, female, athymic nude mice were treated with RC_16_ by i.v. or p.o.at doses of 1 mg/kg and 2 mg/kg, respectively. At 2, 4, 6, 12, 24 and 48 h post RC_16_ administration, a single mouse was anesthetized in an induction chamber with 5.0% isoflurane and an O_2_ flow rate of 0.8 L/min. Each mouse was transferred to a nose cone supplying 5.0% isoflurane with a O_2_ flow rate of 0.8 L/min. After confirming sedation by testing hind footpads, each mouse was subjected to exsanguination by cardiac puncture. Blood samples were collected in BD Microtainers (BD, Franklin Lakes, NJ). Plasma was separated by centrifugation at 2000×*g* for 10 min at 4°C and stored at −80°C until analysis. The RC_16_ concentration in plasma was determined by liquid LC-MS/MS.

### Biodistribution Study

A pilot biodistribution study using one animal per timepoint was performed to minimize the number of experimental animals. Female athymic nude mice were subcutaneously implanted with 5.0 × 10^6^ CHLA-20 cells. When tumors became palpable (approximately 5 mm in diameter), mice were treated with i.v. injections of RC_16_ labelled with Cell-Vue Maroon (dye:RC_16_, 1:100 mol:mole) at a dose of 1 mg/kg. At 12, 24 and 36 h post RC_16_ injection, drug biodistribution was determined using the IVIS-200 (PerkinElmer, Waltham, MA) with filter sets at 760/800 nm (excitation/emission). The mice were then humanely euthanized by CO_2_ asphyxiation. Organs were removed, weighed and used for quantitative optical imaging by the IVIS system.

### *In Vivo* Efficacy Study

#### Xenograft Models

Female athymic nude mice were subcutaneously injected with 5 × 10^6^ human cancer cells in 150 μL mix of PBS and matrigel (2:1). The mice were then randomized into groups of six animals for each tumor type. This number was chosen because we sought a large effect size and to minimize numbers of mice. When tumors reached a mean volume of 150 mm^3^, the animals were treated with RC_16_ or vehicle alone (PBS), given slowly through the tail vein at the dose of 1 mg/kg, 3 times a week for 3 weeks or orally gavaged at the dose of 2 mg/kg/day for 3 weeks.

#### Immunocompetent Model

An efficacy study was performed on an immunocompetent model of neuroblastoma. In this experiment A/J mice were i.v. injected with Neuro 2A (0.2 × 10^6^ cells in 100 μL of PBS). After 5 days, the mice were randomized (six animals per group) and treated once with RC_16_ injected through the tail vein at doses of 20 μg or 40 μg/mouse or vehicle (PBS). After treatment animals were monitored for survival and endpoint criteria.

#### Endpoint Criteria

Endpoint criteria included tumor volume > 2000 mm3, body weight loss ≥ 20%, unusual mouse behavior, lack of movement and poor posture. Tumor size was measured using digital calipers on alternate days and tumor volume was calculated using the following formula: a x b^2^ π/6 where a is the longest diameter and b is the shortest diameter. Mice were also weighed and observed 3 times per week for signs of endpoint condition. Mice that demonstrated signs of toxicity or reached endpoint criteria were humanely euthanized by CO_2_ asphyxiation.

### *In Vitro* Cell Proliferation Assays

Cells were plated in 96-well tissue culture plates at a density of 1 × 10^3^ cells/well, allowed to attach 24 h, and then left untreated or treated with growth medium containing different concentrations of the tested RCn compounds previously dissolved in PBS. After different time periods the cell vitality was determined by MTT assay according to the manufacture’s instruction (Promega). Results are reported as the micromolar concentration of RCn reducing cell survival to 50% (IC_50_).

### Western Blot Analysis

Cells with or without RC_n_ treatment were washed with PBS and lysed on ice for 30 min in lysis buffer containing protease and phosphatase inhibitors. Protein concentrations were determined with the Bio-Rad protein assay kit. 50 μg of total protein was separated on 12% SDS-PAGE at 100 V for 1 h and then transferred onto a nitrocellulose membrane using a wet blotting apparatus (Bio-Rad Laboratories) at 20 V overnight. Proteins were detected by enhanced chemiluminescence detection reagents (Amersham Biosciences). The antibodies used for immunoblotting were: caspases 3, 8, 9, and PARP were diluted 1:100 in blocking reagent (Cell Signaling Technology). The exposure time was the same for all the antibodies. Blots were stripped and reprobed with anti-β-tubulin diluted 1:10,000 in blocking reagent (Santacruz Biotechnology) used as the loading control.

### Caspase Activity Assays

Detection of caspase activity was evaluated by ApoFluor Green Apoptosis Detection kits specific for: caspase-1 and caspase-4; caspase-2, caspase-3 and caspase-7; caspase-6, caspase-8, caspase-9, caspase-10, and caspase-13 (MP Biochemicals), according to the manufacturer’s instructions. Briefly, 5000 cells in 96-well microplates were detached with EDTA and centrifuged at 400 × g for 5 min at room temperature. Cell supernatants were removed, and the pellets resuspended in a buffer containing the appropriate caspase-specific fluorescent probe. After 1 h incubation samples were washed and analyzed by flow cytometry (FACScan, Becton Dickinson) equipped with a 15-mW argon ion laser at 488 nm (16).

### Flow Cytometric Analysis

SH-SY5Y, A673 and 143.98.2 cells were stained with Annexin V-FITC/7AAD according to the manufacturer’s instructions (Biosciences). Briefly, adherent cells were seeded in 6 multi wells at a concentration of 4.5 × 10^5^ cells/well and after 24 h treated with 5 μM RC_16_ for15 h and 24 h. At the end of the treatment the cells were detached, washed twice with cold PBS and then suspended in annexin binding buffer containing 5 μl of Annexin V-FITC and 5 μl of 7AAD. The samples were gently vortexed and incubated 15 min at 25°C in the dark. Finally, 400 ul binding buffer were added to each tube and the samples were analyzed by BD LSR II (Biosciences). The analysis was performed by FlowJo software version 10.0.5.

### Measurement of Intracellular ROS

Intracellular ROS were measured by OxiSelect™ Intracellular ROS Assay Kit (Cell Biolabs) following the manufacturer’s instructions. Briefly, cells (1 × 10^5^/mL) were washed twice in PBS and incubated with 5 μM 2’,7’-dichlorodihydrofluorescein diacetate (H_2_DCFDA) 1 h at 37°C. After PBS washes, the medium was changed with a new one containing 5 μM RC_16_ for 15 h and 24 h. At the end of the treatment samples were washed with PBS and a mixture of medium and cell lysis buffer (1:1) was added for 5 min at 37°C. The fluorescence was read by a fluorimetric plate reader (Spectra max M2, Molecular Device). Excitation wavelength was 480 nm and emission wavelength was 530 nm.

### Electron Microscopy

#### TEM Studies

TEM studies were carried out on SH-SY5Y cells and HFs incubated with 5 μM RC_16_ for 24 h. Cells were seeded on cover glasses for 24 h and then the medium was replaced with a fresh one containing 5 μM RC_16_ for 24 h. At the end of the treatment the samples were washed with PBS and fixed with 2.5% glutaraldehyde in 0.1 M phosphate buffer for 2 h at 4°C and subsequently post-fixed with 1% OsO_4_ in 0.1 M phosphate buffer for 1 h at room temperature. After several washes the samples were dehydrated in an acetone series (70, 90, 100%) and embedded in Epon resin (Fluka, Sigma-Aldrich). Thin sections were collected on nickel grids, stained with uranyl acetate and lead citrate, and observed under a TEM (Philips CM10; FEI). Images were recorded using a Megaview III digital camera (FEI). TEM was also used to prove the formation of micelles in an aqueous environment due to the amphiphilic nature of the RCn molecules. To this purpose an aqueous solution of RC_16_ (20 μM) was stained with 2% (w/v) phosphotungstic acid for 3 min on a copper grid and subsequently it was visualized under TEM.

#### SEM Studies

SH-SY5Y cells were seeded on holders for 24 h and then the medium was replaced with a fresh one containing 20 μM RC_16_ for 1 h. At the end of the treatment the samples were washed with PBS and fixed for SEM in 2.5% glutaraldehyde, then dehydrated in an ethanol series (70, 90, 100%) and critical point dried. The dried specimens were sputter-coated with carbon and examined by FESEM (JSM 890, Jeol). The aqueous solution of RC_16_ (20 μM) was desiccated at RT on the sample holder, coated with carbon and examined by FESEM.

### Interaction of RC_16_ with Sialic Acid

Neuraminidase was used to cleave the sialic acid residues from the polysialilated glycoproteins and gangliosides of the tumor cell surface and the anticancer activity of RC_16_ was subsequently tested in comparison with the cells not exposed to Neuraminidase. Cells were seeded on 96 well plates at a density of 2 × 10^4^ cells /plate and after 24 h were exposed to 50 IU/mL Neuraminidase Type VI from Clostridium Perfrigens (Sigma) for 3 h. Cells were washed 3 times with PBS and afterwards treated with RC_16 ._ The effect on cell number was evaluated after 24 h by MTT assay in comparison with cells not treated with Neuraminidase. The RC_16_ –sialic acid interaction was also evaluated by adding sialic acid to the culture medium in stoichiometric amount with respect to RC_16_. In this experiment cells seeded on 96 well plates at a density of 2 × 10^4^ cells /plate and after 24 h were treated with a mixture of RC_16_ : sialic acid (3 μM : 3 μM). After 24 h the effect on cell number was evaluated by MTT assay in comparison with cells treated with pure RC_16_ or pure sialic acid at the same concentration (3 μM) of the mixture.

### LC-MS/MS Analysis

Chromatographic separation was achieved using a Thermo Scientific Accela UHPLC system with HTS PAL autosampler and an Agilent ZORBAX Extend C18 column (50 × 2.1 mm I.D, 3.5 μm particle size) with a C18 guard cartridge maintained at 35°C. A 6.0 min linear gradient program with water/0.3% formic acid as mobile phase A and acetonitrile/0.3% formic acid as mobile phase B at a constant flow rate of 400 μL/min was applied for elution: 0.0 min 25% B, 1.0 min 25% B, 3.0 min 95% B, 4.5 min 95% B, 4.6 min 25% B and 6.0 min 25% B. The autosampler sample draw was set to 4°C, and a divert valve was used (0.0–2.0 min) to prevent early eluting compounds from entering the MS. Selective reaction monitoring with unit resolution was used for MS detection under positive mode. Transition channels from the protonated molecular ion to selected product ion were 377.33 > 153.15 for RC_16_ and 251.20 > 153.15 for internal standard RC_7_, respectively. Parameters optimized with a direct infusion of analyte and IS using a syringe pump were as follows: spray voltage, 5000 V; sheath gas and auxiliary gas, 25 and 15 (arbitrary unit), respectively; capillary temperature, 325°C; collision gas pressure, 1.5mTorr and skimmer offset, 10 eV. Tube lens offset was 100 eV for both RC_16_ and RC_7_, respectively. Scan time was 0.03 s. Collision energies were 40 eV for RC_16_ and 33 eV for RC_7_. Thermo Scientific LCQuan software was employed for system control and data processing.

### Ability of the RCn Micelles to Encapsulate Bioactive Molecules

Aqueous solutions of RC_16_ (10 mM, 1 mL) were mixed with ethanol solutions of Doxorubicin, Paclitaxel or Etoposide (1 mM, 1 mL). After stirring 12 h at 25 C the mixtures were dialyzed through 5000 Da MW cutoff dialysis membranes against 100 ml water by changing the external medium every 8 h for 3 days. The RC_16_-drug complex collected after dialysis was freeze-dried and the solid residue obtained was spectrophotometrically analysed to estimate the percentage of drug in the final product. The anticancer activity of each complexed drug was evaluated in CHLA-20 as the difference between the activity of the complex and the pure RC_16_ at the same concentration of the complex.

### Statistical Analysis

For the *in vitro* assays, all data points represent mean ± SD, *n* = 6 wells. All the experiments were repeated at least 3 times. Representative data are shown. Statistical significance among the mean values was analysed via an unpaired two-tailed Student *t*-test assuming equal variance. For *in vivo* efficacy studies, *n* = 6 animals. Six was determined to be the minimal number of animals necessary to be able to detect a large effect size. For relative tumor volume (RTV), statistical significance among the mean values was analysed via an unpaired two-tailed Student *t*-test assuming equal variance. Survival statistical significance was determined using the Log-Rank (Mantel-Cox) test on Graph Pad Prism (La Jolla, CA). The significance (p-value) was set at the nominal level of 0.05 or less.

## Results

### Synthesis of the Amphiphilic Amines

**Fig. 1:**
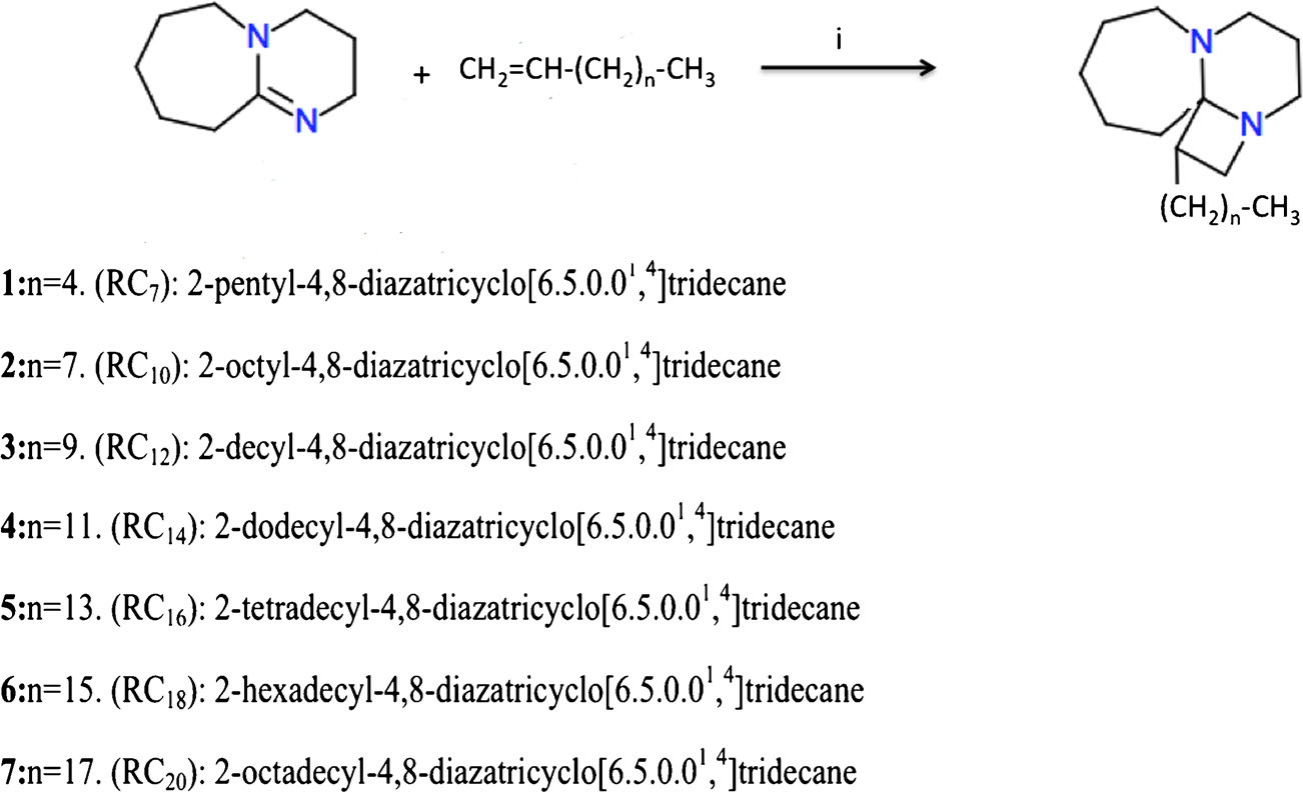
Schema for the synthesis of RCn compounds. (i) N-methylpyrrolidone (NMP), 2-[2-(ethenyloxy)ethoxy]ethan-1-ol CH_2_ = CH-(O-CH_2_CH_2_)_2_-OH as a catalyst, 4 h, room temperature.

The amphiphilic amines were synthesized following the scheme described in Fig. 1. A mixture of 1,8-diazabicyclo[5.4.0]undec-7-ene (DBU) and the appropriate alkene (in a 1:3 Molar ratio) in N-methylpyrrolidone was stirred at room temperature 4 h in the presence of 2-[2-(ethenyloxy)ethoxy]ethan-1-ol 0.2 M as a catalyst. Addition of diethylether induced the precipitation of a solid residue that was purified by flash chromatography using a mixture of methanol/water (9:1) as eluent. The final products were characterized by ^1^H NMR, ^13^C NMR, mass spectra, and elemental analysis (Supplemental Material).

### *In Vitro* Cell Killing

**Fig. 2:**
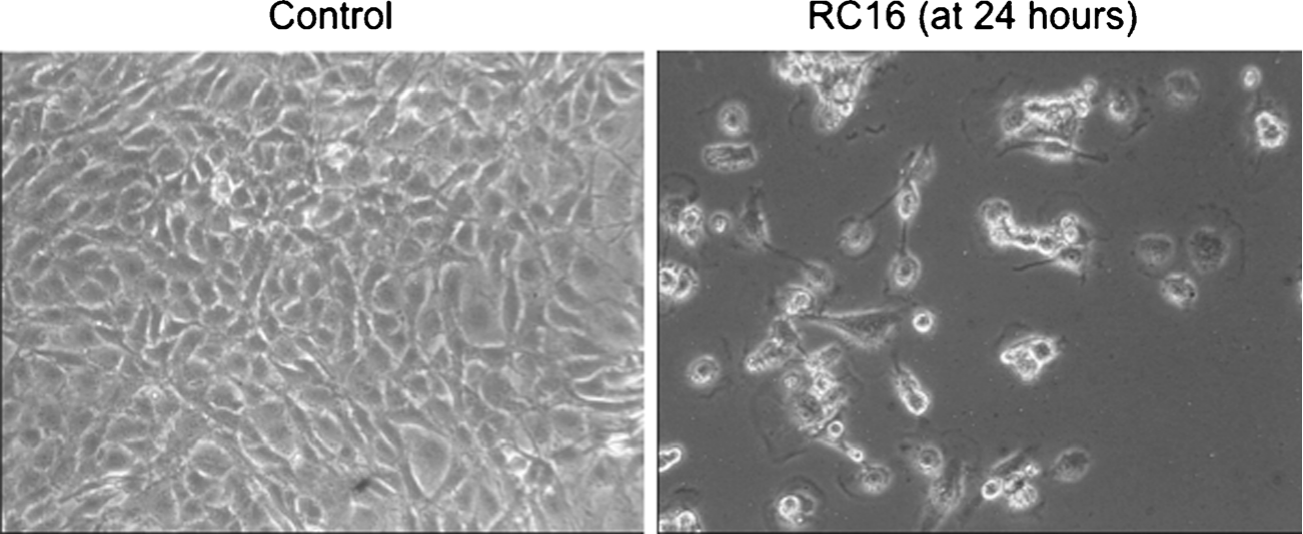
Light microscopy analysis of 143.98.2 tumor cells treated for 24 h with 5uM RC16. The cells appeared reduced in the number, rounded in shape and almost detached from the plastic surface of the flask. All these morphological features are consistent with cell death (magnification 10×). Similar findings were observed in all other cancer cell lines treated with RC16.

Time-dependent and dose-dependent cell killing was observed in the presence of all the RCn compounds of the series (Table I). Differences in activity were obtained among the different RCn with RC_16_ resulting the most active against all the pediatric tumor cell lines analyzed (Table I). Under light microscopy, cells showed characteristic morphologic changes consistent with cell death (Fig. 2). Due to its improved antitumor activity, RC_16_ was further evaluated on a set of adult tumor cell lines (A549, MDA-MB-231, WiDr) selected for being particularly resistant to the current antitumor treatments. RC_16_ was very active with IC_50_ values in the low micromolar range (1–10 μM, Table II). The evaluation RC_16_ on the normal cell lines demonstrated, on the contrary, very low potency with IC_50_ values for human keratinocytes, human fibroblasts and HUVEC more than tenfold higher than those for tumor cells (Table II).

**Table I:** Cell killing activity of RCn compounds on pediatric tumor cell lines (IC50 (μM)) at 24 and 48 h*

Cell Line	Tissue Type	RC_7_		RC_10_		RC_12_		RC_14_		RC_16_		RC_18_		RC_20_	
		24 h	48 h	24 h	48 h	24 h	48 h	24 h	48 h	24 h	48 h	24 h	48 h	24 h	48 h
A673	Ewing’s Sarcoma	5.73	4.11	2.60	2.02	1.82	1.65	1.27	1.13	0.80	0.65	1.03	0.98	1.22	1.35
143.98.2	Osteosarcoma	4.51	3.78	2.55	1.68	1.98	1.24	1.31	0.78	0.50	0.32	0.92	0.65	1.09	0.83
SK-N-AS	Neuroblastoma	5.21	3.43	2.14	1.56	1.87	0.71	1.20	0.63	0.88	0.67	1.23	0.75	1.60	0.90
S462TY	Malignant Peripheral Nerve Sheath Tumor	5.62	3.90	2.39	1.78	2.00	1.09	1.81	0.95	1.11	0.76	1.67	0.88	1.83	1.16
Ramos	Lymphoma	4.55	3.12	2.10	1.03	1.18	0.74	0.96	0.22	0.10	0.04	0.83	0.17	0.97	0.20
Rh41	Rhabdomyosarcoma	5.78	4.01	2.85	2.03	1.75	1.16	1.36	0.97	0.25	0.15	1.01	0.82	1.68	1.12

* Data are representative of three independent experiments

**Table II:** Cell killing activity of R-C_16_ on adult tumor and normal cell lines (IC50 (μM)) at 24 and 48 h)*

**Adult Tumor Cell Line**	**24 h**	**48 h**
A549	6.62	5.58
MDA-MB-231	2.96	1.67
WIDr	7.30	6.02
**Normal Cell Lines**	**24 h**	**48 h**
Human Foreskin Keratinocytes (differentiated)	15.20	13.46
Human Gingival Fibroblasts	11.80	10.50
HUVEC	10.63	9.65

*Data are representative of three independent experiments

### Induction of Apoptosis

**Fig. 3:**
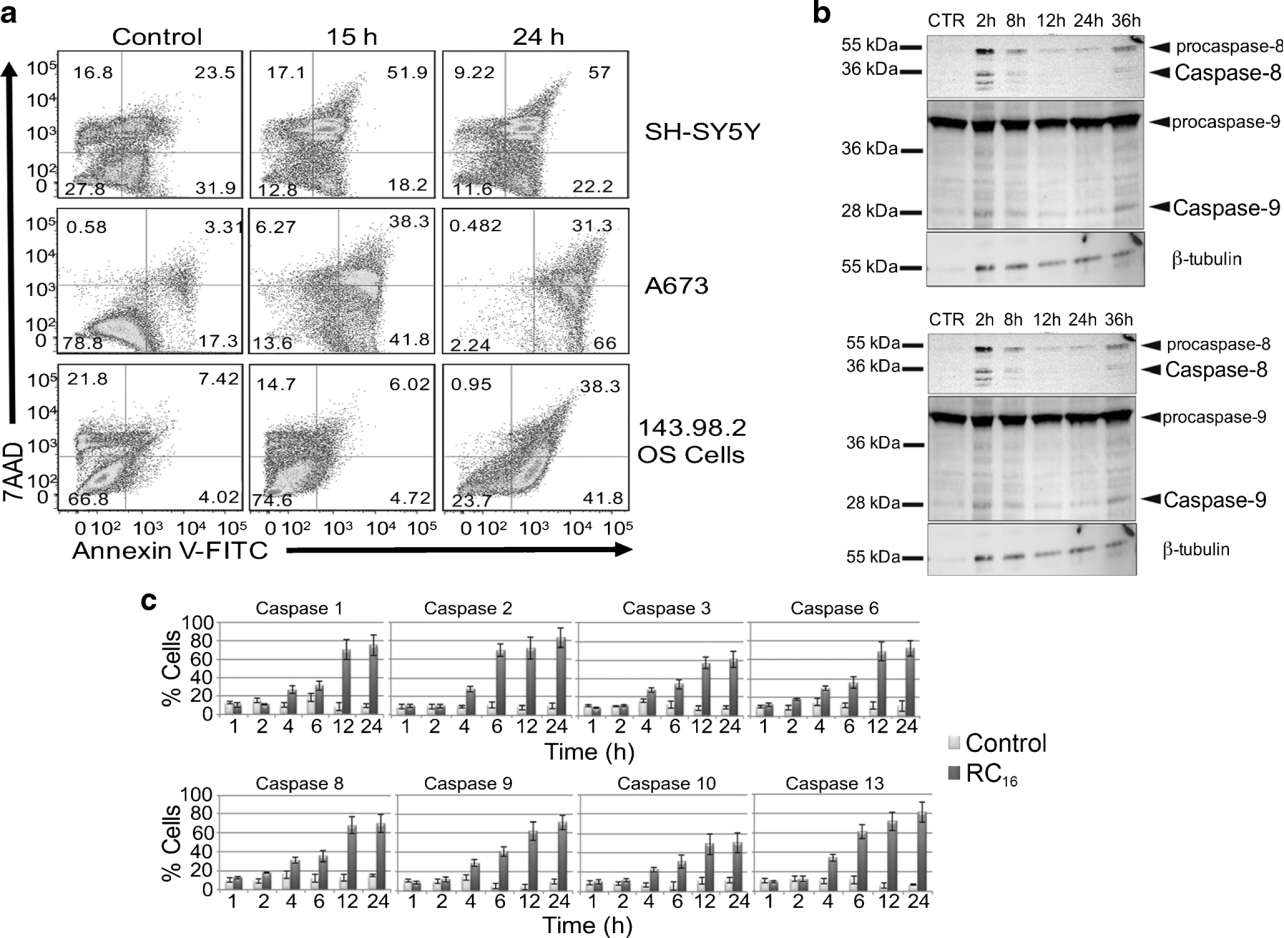
Induction of Apoptosis by RC_16_. (**a**) Annexin V-FITC/7ADD stained fluorescence-activated cell sorter (FACS) in SH-SY5Y, A673 cells and 143.98.2 cells. All the samples were treated with 5 μM RC_16_ for 15 and 24 h. (**b**) Western blot analysis of SH-SY5Y cells treated with 5 μM RC_16_ for 2, 8, 12, 24 and 36 h for caspase 3, 8 and 9. H3h, H9h: Hela treated with 5 μM staurosporine for 3 and 9 h (positive control of apoptosis); S9h: SH-SY5Y treated with 5 μM staurosporine for 9 h (positive control of apoptosis). (**c**) Fluorimetric assay of caspases 1, 2, 3, 6, 8, 9, 10, 13 activation in SH-SY5Y cells treated with RC_16_ 5 μM for 1, 2, 4, 6, 12 and 24 h (*p* < 0.05).

We tested the ability of RC_16_ for the induction of apoptosis. As shown in Fig. 3a control and treated cells were gated into LR (Lower Right), UR (Upper Right), LL (Lower Left) and UL (Upper Left) quadrants. Cells in LR and UR were considered as early apoptotic (annexin+/7AAD-) and late apoptotic (annexin+/7AAD+) respectively. Cells in LL and UL were considered live (annexin-/7AAD-) and necrotic (annexin-/7AAD+), respectively. Flow cytometric analysis showed that the percentage of cells in apoptosis increased over time with differences among the different cell lines. At 15 h the extent of apoptosis expressed as the sum of the percentages in LR and UR quadrants was higher in A673 and lower in 143.98.2. After 24 h all the cells showed approximately 100% apoptosis. These data indicate the apoptotic mode of cell death in all the cell lines analyzed and a different rapidity of cell death depending on the cell type. Differences between control and treated cells are statistically significant at any time of treatment (*p* = 0.016 vs control). To further confirm the apoptotic nature of cell death induced by RC_16_ in tumor cells, we also evaluated whether caspases were involved in the death process. We performed both western blot analysis and fluorimetric assays on SH-SY5Y cells in the presence of RC_16_ . The western blot analysis indicated activation of caspases 3, 8 and 9 after exposure to RC_16_ (Fig. 3b), suggesting the induction of both extrinsic and intrinsic apoptosis. In the fluorimetric assays we used different commercially available kits for single caspases (*i.e*., caspase-1, caspase-2, caspase-3, caspase-6, caspase-8, caspase-9, caspase-10, and caspase-13) and measured the increase in fluorescence after 1, 2, 4, 6, 12, and 24 h in SH-SY5Y cells exposed to RC_16_. As shown in Fig. 3c all the above caspases were activated already at 4 h and became more active thereafter in treated tumor cells with respect to controls.

### Electron Microscopy Analysis of RC16 Treated Cells and the Encapsulation Ability of RC_16_ Micelles Towards Bioactive Molecules

**Fig. 4:**
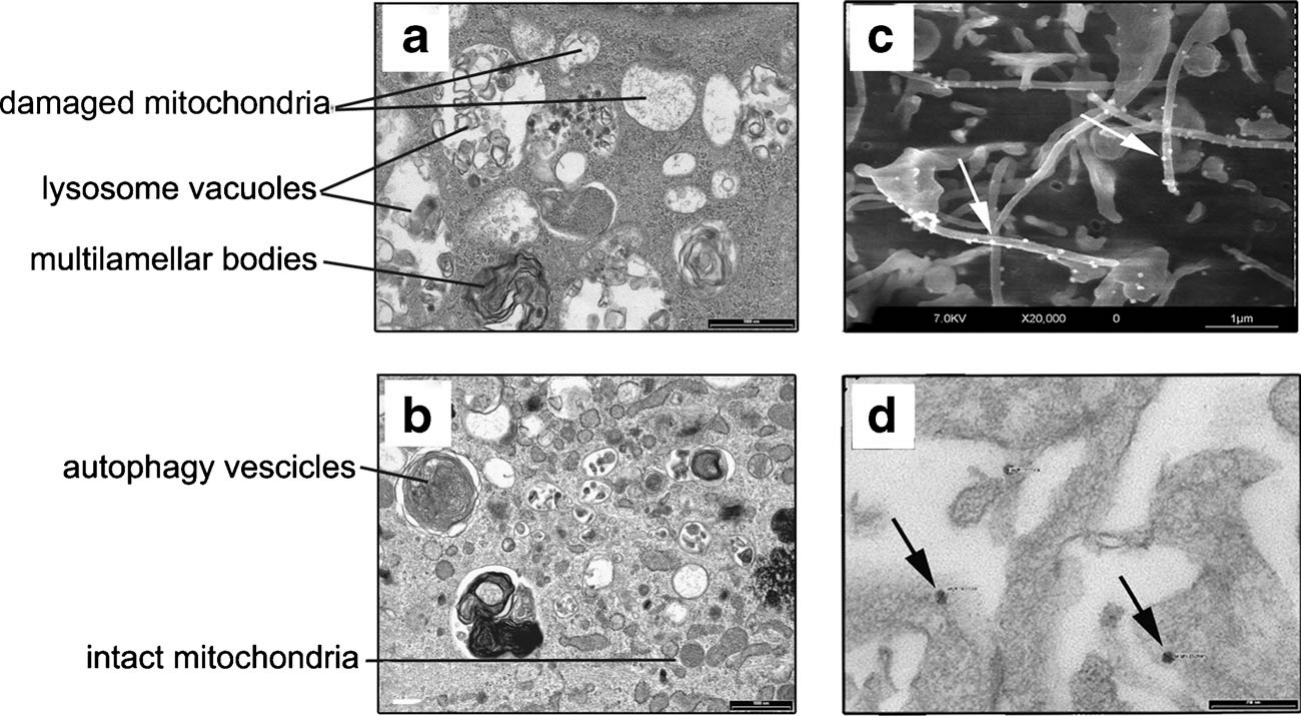
Electron microscopic analysis of RC_16_ treated neuroblastoma cells. (**a**) TEM image of SH-SY5Y cells treated with RC_16_ at 24 h showing damaged mitochondria, multilamellar bodies and lysosomes (bar: 100 nm). (**b**) TEM image of human fibroblasts treated with RC_16_ 5 M at 24 h showing a preserved morphology with intact mitochondria and vesicles correlated with autophagy (bar: 100 nm). (**c**, **d**) Electron microscopy images of RC_16_ micelles formed by the spontaneous self-assembling of the amphiphilic RC_16_ in the presence of SH-SY5Y cells with 20 μM RC_16_. SEM image(C, bar: 1 μm), TEM image (D, bar: 200 nm). Arrows indicate the presence of micelles on the cell surface.

SH-SY5Y cells treated with RC_16_ for 24 h showed a polygonal morphology in which nucleus and nucleolus were well preserved while cytoplasm was characterized by several vacuoles of different diameters (Fig. 4a). At higher magnification damaged mitochondria with no clear cristae were easily detected as well as late autophagy vacuoles (lysosome like vacuoles) with materials at different stages of degradation. Damaged rough endoplasmic reticulum and multilamellar bodies connected with phospholipid degradation were also observed (Fig. 4a). Human fibroblasts treated with RC_16_ at the same concentration and duration showed a fibroblastic morphology and a nucleus well preserved (Fig. 4b). Several small vacuoles were detected in the cytoplasm. Normal mitochondria were observed, while some autophagy vacuoles connected with an early process were detected (Fig. 4b). The formation of micelles, triggered by the spontaneous self-assembling of the amphiphilic RC_16_ molecules in water, was confirmed by Electron Microscopy. SEM and TEM images of RC_16_ in the presence of SH-SY5Y cells displayed micelles of spherical shape and regular surface (Fig. 4c, d). Measure of their mean diameter, performed by TEM, provided values in the nanometric range (20–30 nm). The RC_16_ micelles were able to encapsulate the antitumor drugs studied. The encapsulation efficiency, calculated as the weight percentage of drug in the final complex, followed the order: Doxorubicin (2.3 ± 0.78%) > Paclitaxel (1.8 ± 0.73%) > Etoposide 1.0 ± 0.59%). The anticancer activity of the complexed drugs resulted in each case higher than the free drugs at the same concentrations of the complex. The highest improvement in anticancer activity with respect to the free drug was observed in the presence of Doxorubicin (Table III).

**Table III:** Cell killing activity of Doxorubicin, Paclitaxel and Etoposide Complexed with R-C_16_*

Complex	% CTR	Free drug	% CTR
RC16-DOXO	11.63 ± 1.29	DOXO	27.62 ± 0.55
RC16-PTX	40.34 ± 3.51	PTX	56.93 ± 4.61
RC16-ETO	35.26 ± 2.08	ETO	65.80 ± 5.76

*CHLA-20 cells were seeded in 96 well plates (2 × 10^4^ cells /plate) and after 24 h they were treated with the complexes (3 μM) or with the free drugs at concentrations corresponding to those present in the complexes. After 48 h, the effect on cell number was evaluated by MTT assay. Cell viability was expressed as a percent of the untreated CHLA-20 cells (CTR). Data are representative of three independent experiments (*p* < 0.05)

### Evaluation of Intracellular ROS and the Interaction of RC_16_ With Sialic Acid

**Fig. 5:**
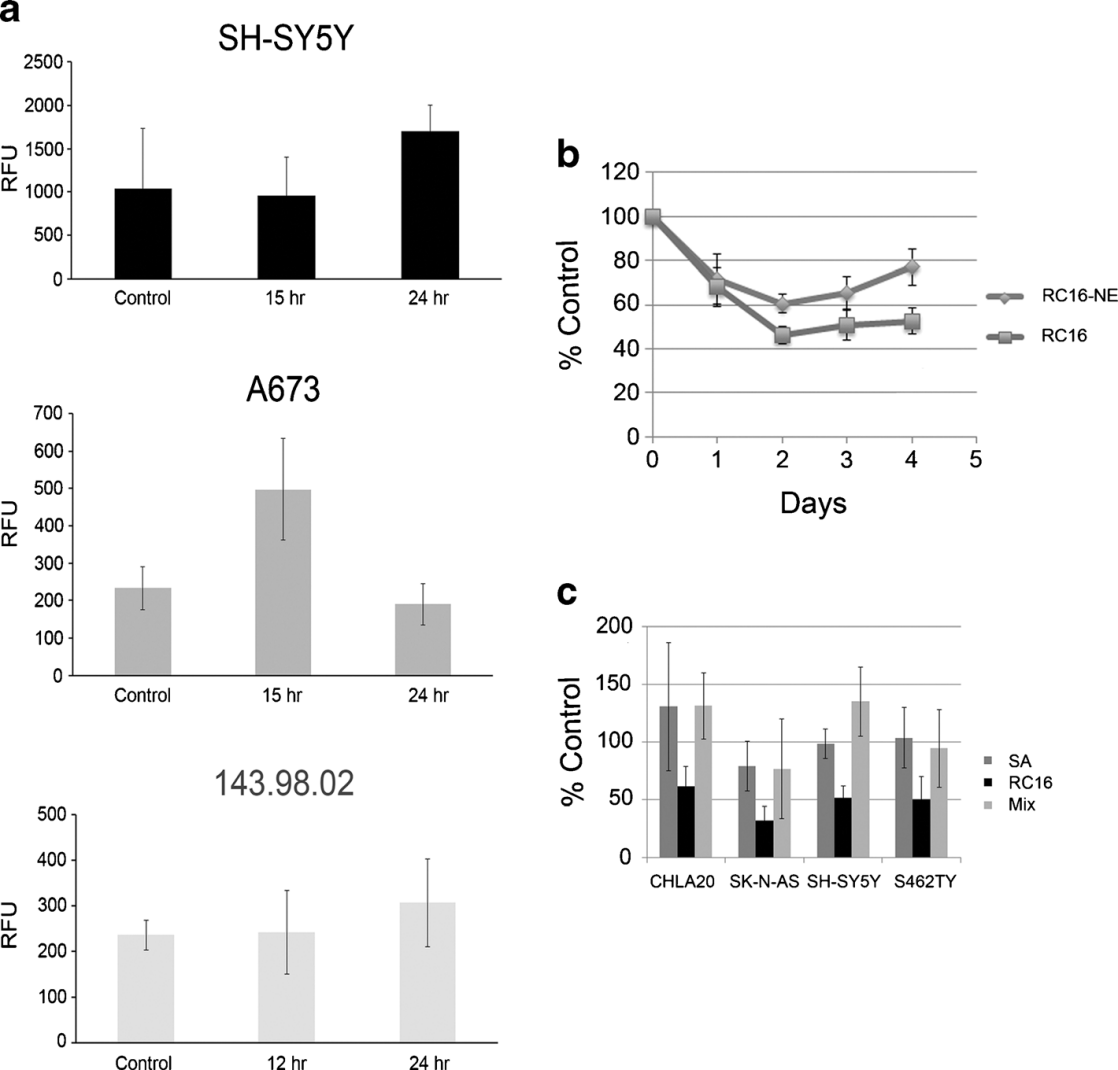
Evaluation of intracellular ROS and the interaction of RC_16_ with sialic acid. (**a**) Measurement of intracellular ROS in A673, 143.98.2 and SH-SY5Y after treatment with 5 μM RC_16_ for 15 h or 24 h. (*p* < 0.05). (**b**) Anticancer effect of RC_16_ (3 μM) on S462.TY cells (RC_16_) in comparison with the same cells previously treated with Neuraminidase (50 mU/ml) (RC_16_-NE). (**c**) Tumor cells treated with RC_16_ (3 μM) or a mixture of RC_16_ + Sialic Acid (3 μM:3 μM) or pure Sialic Acid (3 μM) for 24 h.

The evaluation of intracellular ROS confirmed the damage to mitochondria observed by electron microscopy. After treatment with RC_16_, intracellular ROS increased in all the tumor cell lines analysed. The highest increase was observed in A673 where the ROS levels became more than twice the control values after 15 h of cell exposure to RC_16_ (Fig. 5a). In SH-SY5Y and 143.98.2 the ROS increase occurred after longer time periods (24 h) of cell exposure to RC_16_ with lower level increase relative to the controls.

The pre-treatment of cells with Neuraminidase, decreasing the presence of sialic acid on the glycoproteins and gangliosides of the cell surface, decreased the anticancer activity of RC_16_ (Fig. 5b) supporting the hypothesis of an interaction of RC_16_ with the sialic acid residue of the cell membrane as a contribution to its whole mechanism of action. As confirmation, the addition of sialic acid to the culture medium, interacting with RC_16_ and therefore decreasing its availability towards the sialic acid residues present on the glycoproteins and gangliosides of the tumor cell surface, decreased the RC_16_ anticancer activity (Fig. 5c).

### Maximum Tolerated Dose in Mice

Prior to initiation of the experiment, animals were naïve to drug and weighed an average of 0.023 kg. The maximum tolerated dose of RC_16_ by intravenous route was 4 mg/kg. At this dose 5/5 (100%) survival was obtained with no body weight loss with respect to the controls. Moreover, no signs of toxicity such as altered mouse behavior, movement or diarrhea were observed. By increasing the dose above 4 mg/kg a progressive decrease in survival was observed with a 0/5 (0%) survival at 5.5 mg/kg.

### Pharmacokinetics and Biodistribution of RC_16_

**Fig. 6:**
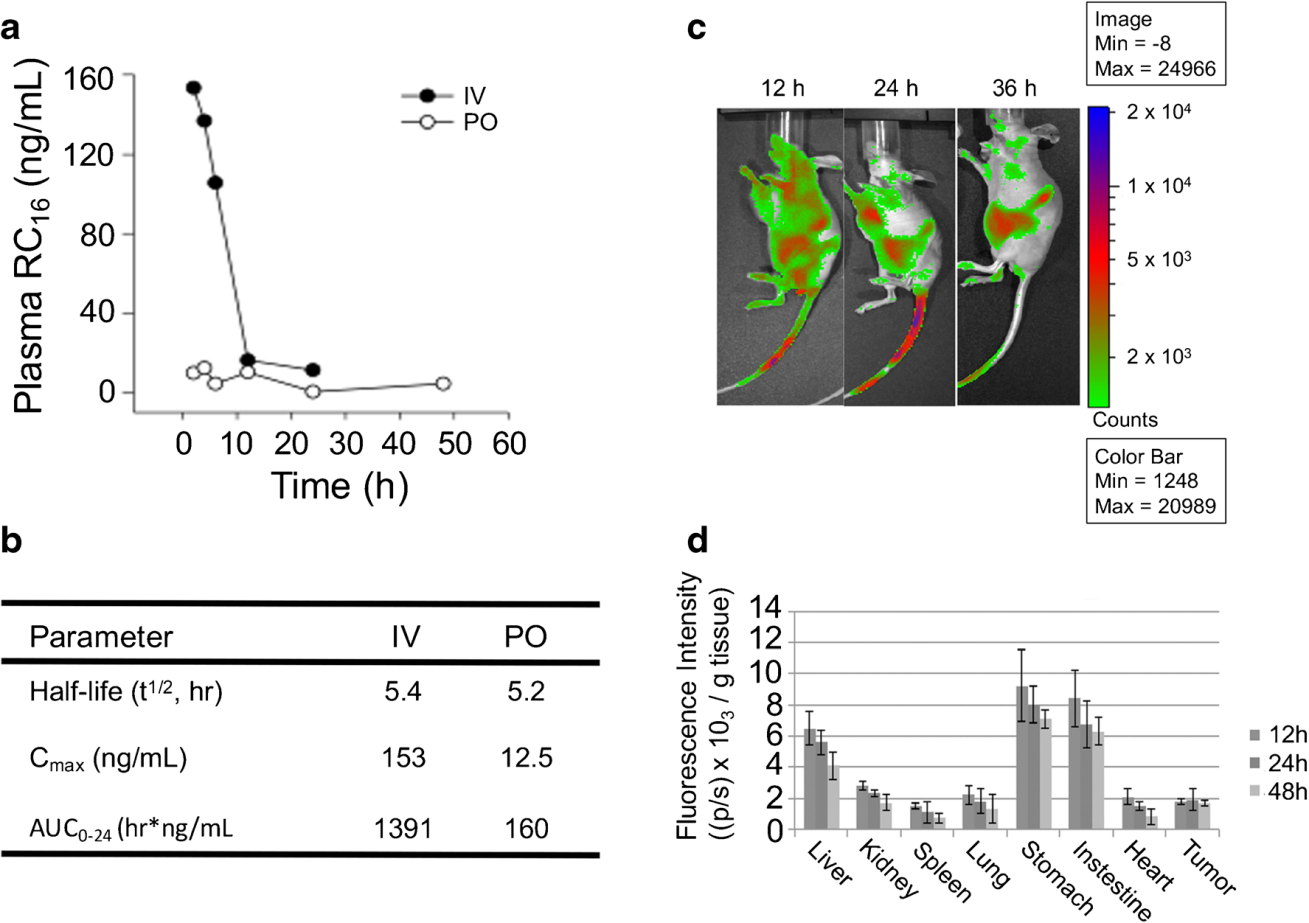
Pharmacokinetic and biodistribution studies of RC_16_ in mice. Plasma concentration-time profiles of RC_16_ after intravenous or oral (**a**) administration at 1 mg/kg or 2 mg/kg respectively (*n* = 1 mouse per time point). (**b**) Non-compartmental pharmacokinetic parameters of RC_16_. t_1/2α_ and t_1/2β_ represent half-lives in the initial distribution phase and in the subsequent elimination phase respectively; C_0_ is the initial drug concentration in blood; V_d_ is the volume of distribution; AUC is the area under the blood concentration *versus* time curve. (**c**) Biodistribution of RC_16_ labelled with Cell-Vue Maroon (dye:RC_16_ 1:100 mol:mole) in nude mice bearing CHLA-20 tumors (*n* = 3). The labelled RC_16_ was injected i.v. at 1 mg/kg and whole-body images of the mice were taken at 12, 24 and 36 h after i.v injection. (**d**) Fluorescence intensity in tissues and organs at 12, 24 and 48 h after the i.v. injection of RC_16_ labelled with Cell-Vue Maroon.

The pilot pharmacokinetic data were obtained after RC_16_ administration at 1 mg/kg in mice by i.v. or 2 mg/kg by oral route. Each data point is from a single mouse. At 2 h post i.v. administration, the plasma concentration was 153 ng/mL with a continued decline to 11.4 ng/mL by 24 h and a measured half-life of 5.4 h (Fig. 6a). After oral administration the maximum observed plasma concentration of RC_16_ was 12.5 ng/mL at 4 h. The concentration continued to decline and was measured at 0.5 ng/mL and 4.6 ng/mL in the plasma samples collected from the mice euthanized at 24 and 48 h, respectively (Fig. 6a). Non-compartmental analysis of these pilot data indicated an apparent half-life of 5.2 h, similar to that from intravenous dosing. The pharmacokinetic parameters of RC_16_ following intravenous and oral administration in nude mice are summarized in Fig. 6b.

Bioluminescence images showed an extensive distribution of fluorescence in the whole body at 12 h and a gradual decrease of distribution at 24 and 36 h with a persistence of fluorescence in the liver and the gastrointestinal system (Fig. 6c). For a quantitative evaluation of biodistribution the organs were removed from the euthanized mice at 12, 24 and 48 h after i.v. injection of the labelled RC_16_ and were imaged immediately after dissection. All images were acquired for 5 s and processed using the Xenogen Living Image software (25). The fluorescence intensity (photons/second, (p/s)) was calculated per unit weight of each organ (26), and the results are shown in Fig. 6d. At each time point stomach, intestine and liver showed the highest fluorescence intensity. All the organs showed a decrease in RC_16_ content over time except the tumors whose RC_16_ level remained constant over time.

### *In Vivo* Efficacy Studies

**Fig. 7:**
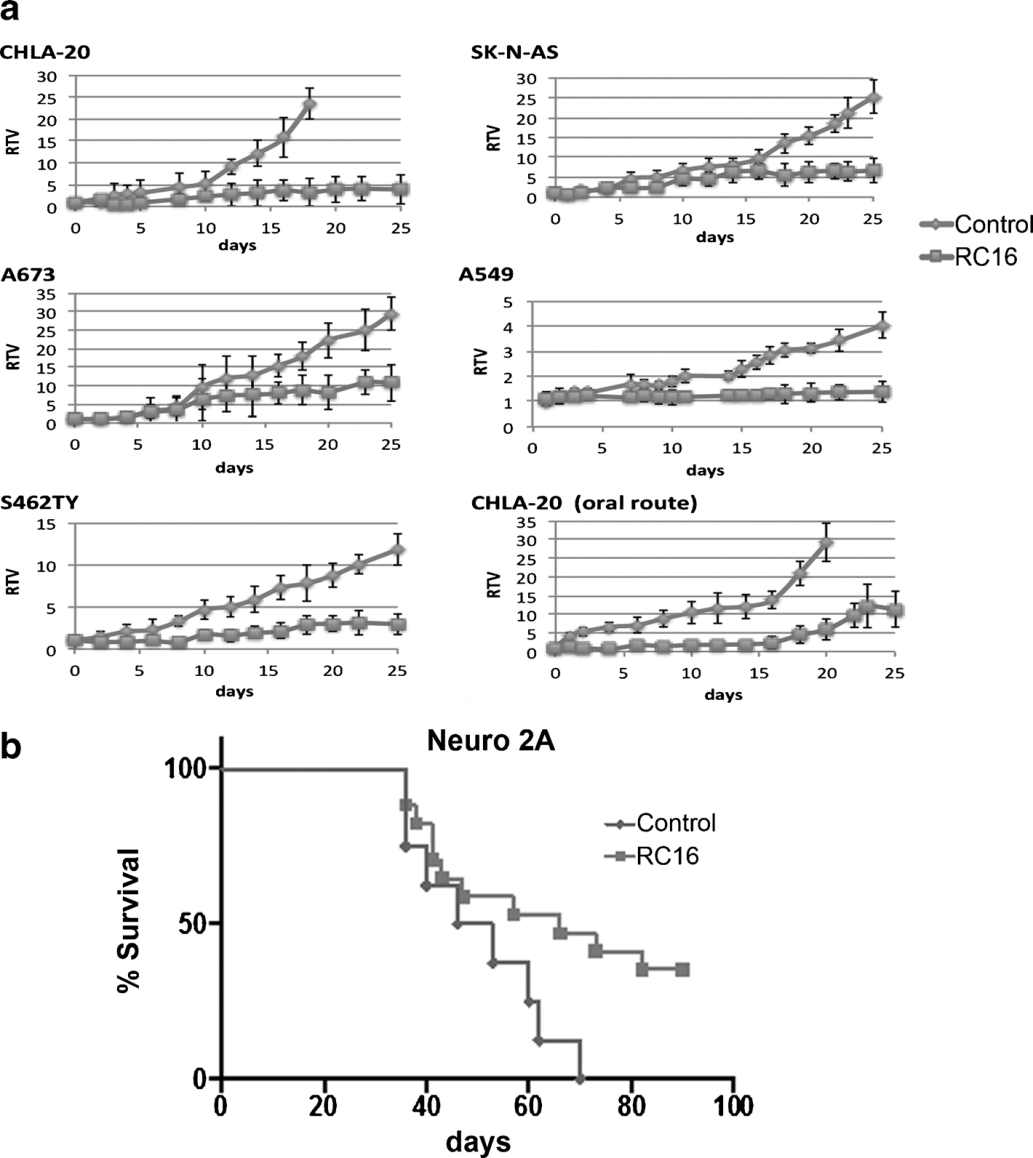
Evaluation of RC_16_ for *in vivo* efficacy. (**a**) Inhibition of the tumor growth in tumor xenograft models by intravenous administration (CHLA-20, SK-N-AS, A673, A549, S462TY) or oral administration (CHLA-20) of RC_16_ . RC_16_ was given through the tail vein at the dose of 1 mg/kg, 3 times a week for 3 weeks or gavaged at the dose of 2 mg/kg/day for 3 weeks. RTV is obtained by TVn/TV_0_, where TVn is the tumor volume at day n and TV_0_ is the tumor volume at day 0) (P-values < 0.05). (**b**) Improvement of the survival in a metastatic model of murine neuroblastoma (Neuro-2A) by intravenous administration of RC_16_. RC_16_ was injected through the tail vein at doses of 20 μg (*n* = 6) or 40 μg/mouse (*n* = 6) (plotted together) (*p*-values < 0.05).

Prior to initiation of the experiment, animals were naïve to drug and weighed an average of 0.023 kg. Administration of RC_16_ to six mice bearing human tumor xenografts in each treatment group demonstrated a strong antitumor activity by both intravenous and oral administration (Fig. 7a). No weight loss or signs of toxicity were observed in any case during the treatment period indicating good tolerability of RC_16_ both by intravenous administration and gavage. In the metastatic model of neuroblastoma disease the mice showed a significant increase in the mean survival time with respect to the controls (Fig. 7b) indicating a strong activity of RC_16_ after a single intravenous administration. At the end of the experiment (90 days) 3 of the 12 RC_16_ treated mice were healthy and alive and showed no evidence of macroscopic disease at necropsy.

## Discussion

We created and tested a novel class of amphiphilic amines (RCn) based on a tricyclic hydrophilic head and a hydrophobic alkyl linear tail of variable length for their antitumor activity and their ability to complex bioactive molecules. We observed anticancer activity of the RCn amines in all of the cancer cell lines analysed, including both pediatric and adult tumors, greater than seen in the normal cells.

RC_16,_ characterized by an intermediate alkyl tail length, exhibited the strongest anticancer activity in the series and was chosen as a lead compound for further study. RC_16_ exhibited significant antitumor activity in several cancer models by both intravenous and oral routes. Mechanistic studies suggest RC_16_ binds to sialic acid residues on the cell membrane and induces cell death via mitochondrial and lysosomal damage. Both activities are likely to account for the differential effects on tumor compared with normal cells. The interaction with sialic acid is an interesting target in cancer research as it enables selectivity for tumor cells due to high levels of the polysialilated glycoproteins and gangliosides on their surface. The interaction of RC_16_ with sialic acid may be attributed to the presence of the 4-membered azetidine ring on its hydrophilic head that links the polysialic acid residues of the tumor cell membranes (27–29). Indeed azetidines are known to easily react with nucleophiles undergoing acid catalyzed ring opening reactions (30–32) due to their high ring strain energy, especially in the presence of substitution like in RC_16_ where the substituent is represented by a cyclic structure. The RC_16_ interaction with polysialic acid is expected to produce structural modifications on the glycoproteins and gangliosides of the tumor cell surface thus inducing perturbations to the functional properties of the cell membrane that, in addition to the other alterations induced in mitochondria and lysosomes, contribute to the final antitumor activity of RC_16_. In normal cells, on the contrary, the very limited expression of sialic acid avoids any significant interaction of RC_16_ with the cell surface.

The molecular mechanisms underlying the observed intracellular effects are not completely clear, but the particular molecular structure of RC_16_ may account for its multiple mechanisms of action. Indeed RC_16_, as the other RCn, is comprised of 4, 6 and 7 membered rings condensed in a unique tricyclic assembly linked to an alkyl linear tail. The hydrophobic cycles surrounding the amine groups in the tricyclic assembly provide a counter-balance to the hydrophilicity increase triggered by the amine groups’ protonation in an aqueous physiological environment thus allowing the molecules to partition through biological membranes in spite of the presence of positive charges. Thus the observed damage to the inner mitochondrial membranes and the increase in the cell ROS levels in the tumor cells may be attributed to an accumulation of RC_16_ in mitochondria. This accumulation might be promoted by both the negative transmembrane potential of the mitochondrial inner membrane (negative inside), which favors passage of protonated molecules into the matrix, and by the acidic pH of the intermembrane space which attracts amine molecules from the cytoplasm inside the mitochondria (14). Moreover, the high content of negatively charged phospholipids such as cardiolipin on the outer mitochondrial membrane might further favor accumulation of protonated molecules on the mitochondrial surface (33). Similarly, the observed alterations of the lysosomes in the tumor cells treated with RC_16_ may be attributed to an accumulation of RC_16_ in the acidic inner environment of lysosomes driven by the same suitable hydrophilic/hydrophobic balance of the molecule allowing its partition across the lysosomal membrane and a further protonation inside (10–13).

In addition to differential expression of sialic acid, we hypothesize that the differential effects of RC_16_ on tumor compared with normal cells are due to differences in organelle membrane potentials. We observed the mitochondria and lysosomes alterations and the ROS increase after treatment with RC_16_ in cancer cells but not in normal cells. This finding is in accordance with the observation that the mitochondria of cancer cells, in contrast to normal cells, are characterized by a very large membrane potential across the mitochondrial membrane (up to 150–160 mV, negative inside) driving an extensive uptake of partitionable amines within the mitochondrial matrix (14–16). Likewise, the lysosomes of cancer cells are characterized by an increase in lysosomal enzymes and a deregulation of the enzymatic functions which, providing acidification defects and metabolic perturbations, may make the lysosomal structure more sensitive to the accumulation of partitionable amines (17,18). The mechanism of cell death induced by RC_16_ appears to be mostly via apoptosis, involving the activation of initiator caspases (caspase-2, caspase-8, caspase-9, caspase-10), executioner caspases (caspase-3, caspase-6) and also pro-inflammatory caspases (caspase-1 and caspase-13). The role of the pro-inflammatory caspases in cell death is likely related to their ability to induce host inflammatory responses *in vivo*, thus their contribution to cell death may be observed *in vivo* but not *in vitro*. While the ability to accumulate into the mitochondria and the lysosomes of tumor cells is present in other amine-type antitumor drugs such as the Delocalized Lipophilic Cations (Rodhamine, MKT-077 F16 and Dequalinium) and the Lysosomotropic Drugs (Chloroquine and MDL 72527) (32,34–37), the ability to interact with sialic acid is unique to RC_16_.

The generalized nature of the effects of RC_16_ may have a distinct advantage over drugs that attack specific molecular targets as it may be more difficult for cells to develop resistance. Cells are able to develop resistance to drugs that bind DNA, RNA or RTKs through the expression of P-glycoprotein, Multidrug-Resistance associated Proteins, activation of alternative RTK pathways, or acquisition of non-binding mutations (7). Down-regulation of sialic acids may be a mechanism whereby cells could escape the effects of RC_16_, though such global dysregulation may not be compatible with the tumorigenic phenotype.

It will likely be possible to further increase the therapeutic efficacy of RC_16_. For example, we did not optimize drug exposure by maintaining constant drug plasma concentrations over time as the well known limitations imposed by the mouse models make it difficult to test a continuous infusion regimen. Without continuous infusion, it is possible to reach plasma drug concentrations in the therapeutic window for limited periods of time followed by decrease in concentration which may allow the tumor mass to resume its growth. RC_16_ distributes to the gastrointestinal tract, liver and kidney due to its amine character which, following protonation, drives the RC_16_ circulating molecules into the distribution pathway of the cationic compounds. In this pathway a major role is played by the organic cation transporters (OCT1 and OCT2) which are highly expressed in basolateral membranes of hepatocytes, intestine enterocytes and renal proximal tubular cells and mediate the elimination of a variety of cationic compounds such as drugs, toxins, and endogenous molecules (38), but whether they play a role in RC16 metabolism is unknown. Although a limitation of the study is that we only tested one mouse per timepoint, the pilot pharmacokinetic data demonstrated we were able to achieve concentrations in the 150 to 200 ng/mL range (approximately 500–600 nM) at the low i.v. dose of 1 mg/kg. Oral dosing with RC_16_ may be worth further study, as we did estimate an oral bioavailability of approximately 6% based on the pilot pharmacokinetic data. We have not determined an MTD for this route of administration, and higher doses that could produce similar or even greater plasma exposures may be achievable via oral gavage.

In addition to its activity as an antitumor drug, RC_16_ has been evaluated for its ability to complex other bioactive molecules such as Doxorubicin, Etoposide and Paclitaxel. The complexation ability of RC_16_ is based on its amphiphilic character triggering a spontaneous self-assembling of molecules in water with formation of micelles. As it is well known that micelles with a hydrophobic inner core and a hydrophilic outer shell may encapsulate hydrophobic or partially hydrophobic drugs (39,40), forming micellar aggregates also referred to as complexes, complexation of poorly water soluble drugs into the micelles may improve their aqueous solubility and consequently their bioavailability and therapeutic efficacy (41–46). Micellar complexation may moreover modify the drug disposition in the body by preventing uncontrolled drug distribution and favoring drug accumulation in solid tumors where the discontinuity of the capillaries and the lack of an efficient lymphatic drainage supports the entrapment and retention of nanoparticulate systems such as micelles, liposomes or soluble macromolecules (39,40,47). The micellar complexation of Paclitaxel and Etoposide is regarded with great interest to improve their bioavailability (48,49) overcoming the adverse reactions associated with the currently marketed formulations. Indeed, apart from Abraxane®, consisting in nanoparticles of Paclitaxel and human serum albumin, all the other marketed formulations of Paclitaxel and Etoposide contain excipients such as Cremophor-EL, ethanol, benzyl alcohol, polysorbate-80 which have been associated with serious side effects (50). The micellar complexation of Doxorubicin is also considered a very interesting approach but in this case the main purpose is a modification of the drug biodistribution in addition to an increase in its solubility. Indeed Doxorubicin induces a strong dose-limiting cardiotoxicity (51), and its encapsulation in micelles or liposomes has proven to decrease its distribution to the heart with a reduction of its cardiotoxic effect (52). Simultaneously, increased solubility by micellar complexation has proven to enhance its therapeutic activity (53).

In summary, we have developed a novel, multimechanistic antitumor drug class that shows promise for cancer therapy. These amphiphilic amines bind to and induce anticancer activity by several mechanisms of action with differential effects on normal cells, resulting in a clinically significant therapeutic window. Furthermore, these agents are useful for encapsulating traditional chemotherapies and thereby enhancing their efficacy and reducing their side-effects.

## Electronic Supplementary Material

Below is the link to the electronic supplementary material.

Figure S1 Synthesis analysis, 1H NMR. (JPG 161 kb)

Figure S2 Synthesis analysis, 13C NMR. (PDF 42 kb)

Figure S3 Synthesis analysis, mass spectra. (PDF 34 kb)

Figure S4 Synthesis analysis, elemental analysis. (DOCX 107 kb)

## Abbreviations

7-AAD: 7-amino-actinomycin D
AAALAC: Association for Assessment and Accreditation of Laboratory Animal Care
ATCC: American Type Culture Collection
DBC: 1,7-diazabicyclo [4.4.0] dec-5-ene
DOXO: Doxorubicin
ETO: Etoposide
FBS: fetal bovine serum
HUVECS: Human Umbilical Vein Endothelial Cells
i.v.: intravenous
IACUC: Institutional Animal Care and Use Committee
IC_50_: half maximal inhibitory concentration
IU: international units
IVIS: *In Vivo* Imaging System
LC-MS/MS: liquid chromatography-tandem mass spectrometry
MTD: maximum tolerated dose
MTT: 3-(4,5-dimethylthiazol-2-yl)-2,5-diphenyltetrazolium bromide
p.o.: oral gavage
PBS: phosphate buffered saline
PTX: Paclitaxel
ROS: reactive oxygen species
RPMI: Rosewell Park Medical Institute-1640 media
RTK: Receptor Tyrosine-Kinases
RTV: relative tumor volume
SDS-PAGE: SDS polyacrylamide gel electrophoresis
SEM: Scanning electron microscopy
TEM: transmission electron microscopy
