# Supplementary figures and images for: Preparation and Evaluation of a Novel Class of Amphiphilic Amines as Antitumor Agents and Nanocarriers for Bioactive Molecules
Source: Pharm Res. 2016 Jul 25;33(11):2722–35. doi: 10.1007/s11095-016-1999-9 (PMC5040747; doi:10.1007/s11095-016-1999-9)

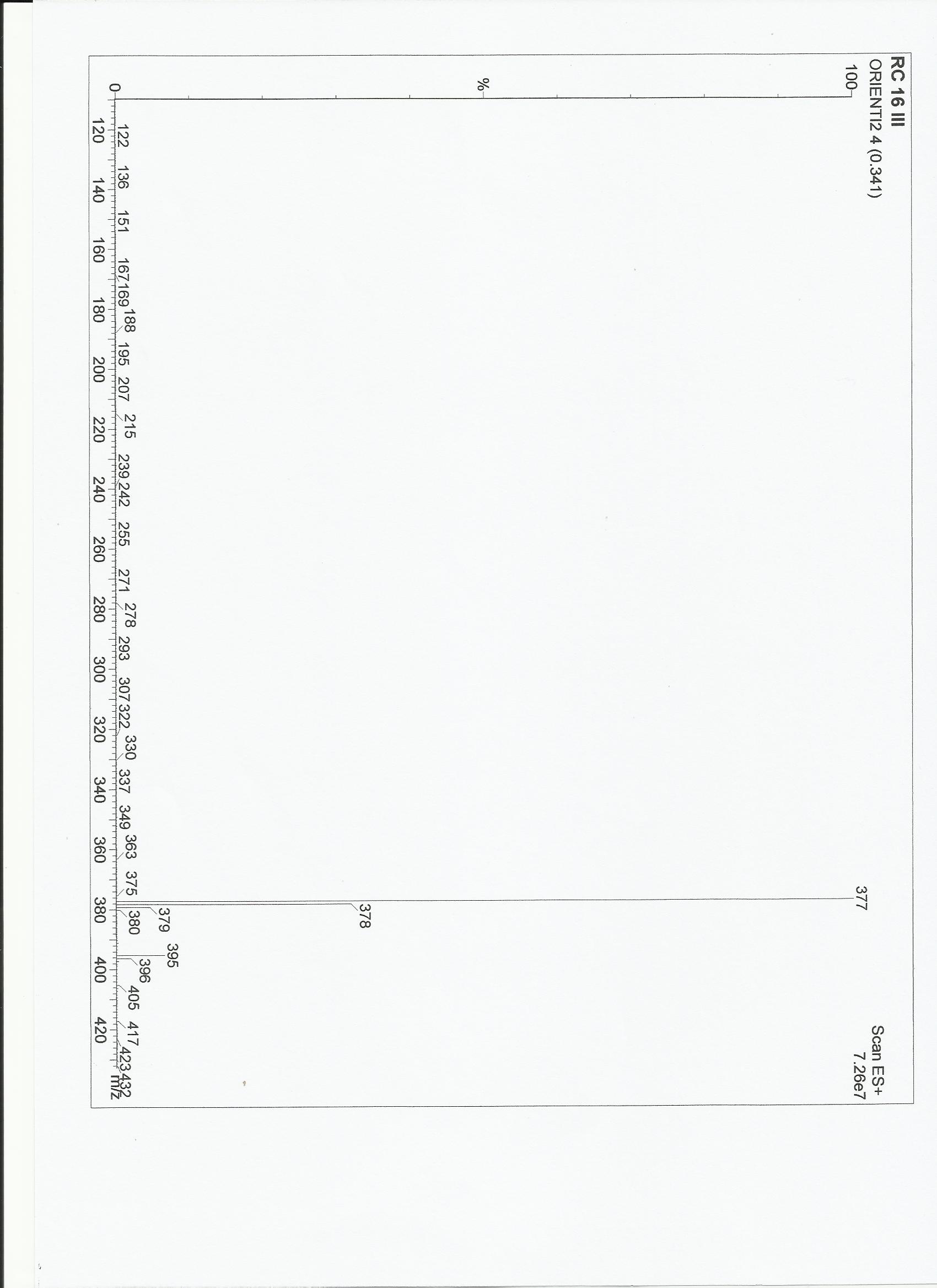

Supplement: Supplementary file 1 — Synthesis analysis, 1H NMR. (JPG 161 kb) [file 11095_2016_1999_Fig8_ESM.jpg]
