# Supplementary material for: Preparation and Evaluation of a Novel Class of Amphiphilic Amines as Antitumor Agents and Nanocarriers for Bioactive Molecules
Source: Pharm Res. 2016 Jul 25;33(11):2722–35. doi: 10.1007/s11095-016-1999-9 (PMC5040747; doi:10.1007/s11095-016-1999-9)

C16.USA.dializzato

Sample Name:
vnmr1
Archive directory:

Sample directory:

FidFile: C16.USA.dializzato.H1

Pulse Sequence: Proton (s2pul)
Solvent: D2O
Data collected on: Dec 22 2011

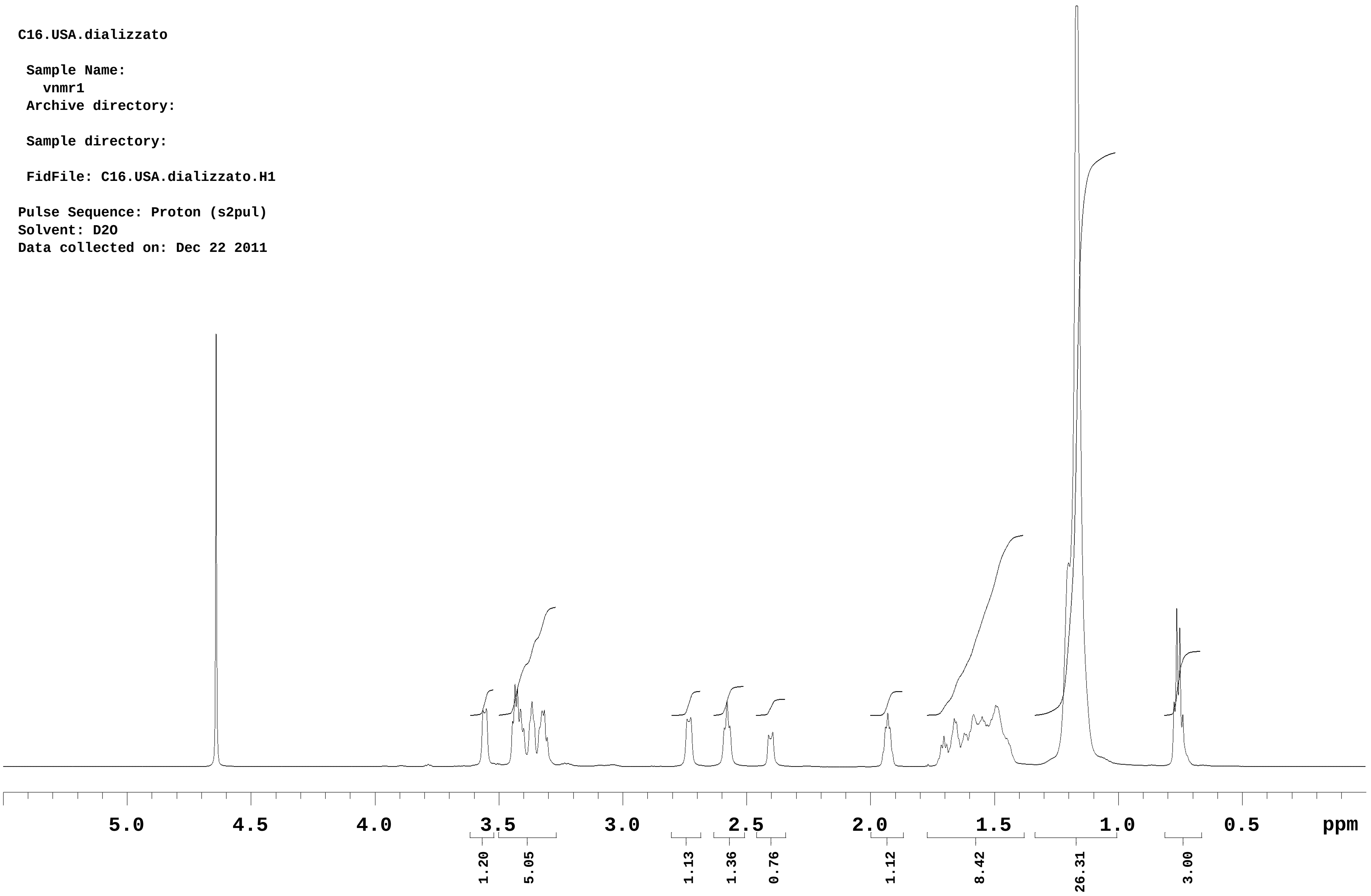

Supplement: Supplementary file 2 — Synthesis analysis, 13C NMR. (PDF 42 kb) [file 11095_2016_1999_MOESM1_ESM.pdf]
