# Supplementary material for: Preparation and Evaluation of a Novel Class of Amphiphilic Amines as Antitumor Agents and Nanocarriers for Bioactive Molecules
Source: Pharm Res. 2016 Jul 25;33(11):2722–35. doi: 10.1007/s11095-016-1999-9 (PMC5040747; doi:10.1007/s11095-016-1999-9)

C16.USA.dializzato

Sample Name:

Archive directory:

Sample directory:

FidFile: C16.USA.dializzato.C13

Pulse Sequence: Carbon (s2pul)
Solvent: D2O
Data collected on: Dec 22 2011

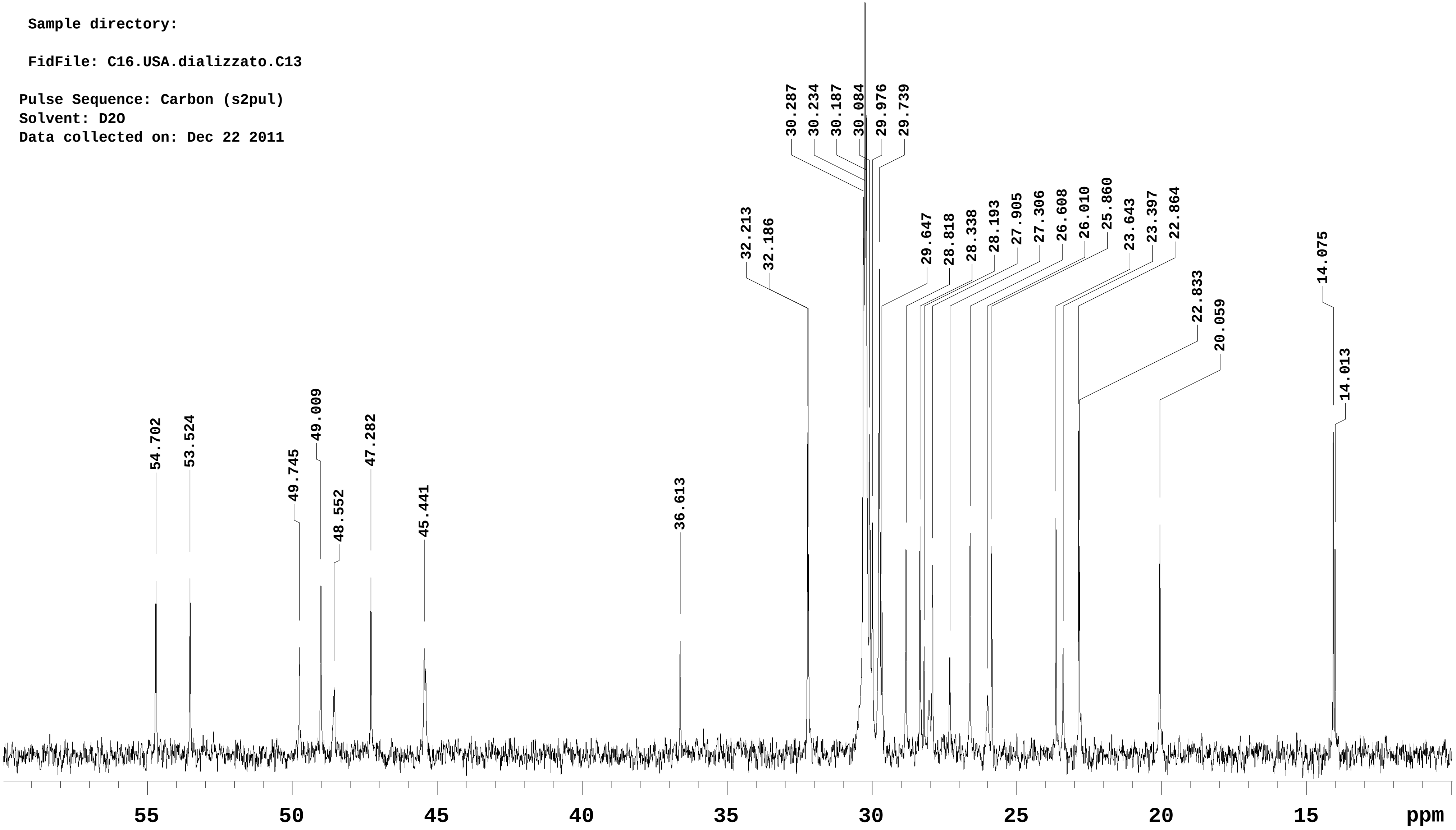

Supplement: Supplementary file 3 — Synthesis analysis, mass spectra. (PDF 34 kb) [file 11095_2016_1999_MOESM2_ESM.pdf]
