# Supplementary material for: Preparation and Evaluation of a Novel Class of Amphiphilic Amines as Antitumor Agents and Nanocarriers for Bioactive Molecules
Source: Pharm Res. 2016 Jul 25;33(11):2722–35. doi: 10.1007/s11095-016-1999-9 (PMC5040747; doi:10.1007/s11095-016-1999-9)
Supplement: Supplementary file 4 — Synthesis analysis, elemental analysis. (DOCX 107 kb) [file 11095_2016_1999_MOESM3_ESM.docx]

Compounds **1-7** were synthesized following the procedure reported in Figure 1 and were characterized by ^1^H NMR, ^13^C NMR, mass spectra, and elemental analysis:

**1:**n=4. (RC_7_): 2-pentyl-4,8-diazatricyclo[6.5.0.0^1^,^4^]tridecane

Pale yellow viscous liquid: 58% yield; ^1^H NMR (D_2_O, 600 MHz): δ 0.79 (t, 3H), 1.21 (m, 8 H), 1.37-1.69 (m, 8H), 1.92 (t, 1H), 2.35 (d, 1H), 2.51 (t, 2H), 2.70 (d, 1H), 3.29-3.46 (m, 5H), 3.57 (t, 1H); ^13^C NMR (D_2_O, 600MHz): δ 14.0, 20.2, 22.9, 28.7, 29.5, 30.1, 32.1, 36.7, 45.5, 47.2, 49.1, 53.3, 54.7 ppm; ESI-MS (m/z): 250 M+:Anal.Calcd for C_16_H_30_N_2_: C 76.74, H 12.07, N 11.19; found: C 76.83, H 12.21, N 11.25.

**2:**n=7. (RC_10_): 2-octyl-4,8-diazatricyclo[6.5.0.0^1^,^4^]tridecane

Pale yellow viscous liquid: 63% yield; ^1^H NMR (D_2_O, 600 MHz): δ 0.70 (t, 3H), 1.22 (m, 14 H), 1.40-1.70 (m, 8H), 1.91 (t, 1H), 2.38 (d, 1H), 2.54 (t, 2H), 2.71 (d, 1H), 3.30-3.43 (m, 5H), 3.57 (t, 1H); ^13^C NMR (D_2_O, 600MHz): δ 14.1, 20.1, 22.9, 28.7, 29.6, 30.0, 32.1, 36.6, 45.5, 47.2, 49.0, 53.5, 54.5 ppm; ESI-MS (m/z): 292 M+:Anal.Calcd for C_19_H_36_N_2_: C 78.02, H 12.41, N 9.58; found: C 78.15, H 12.10, N 9.62.

**3:**n=9. (RC_12_): 2-decyl-4,8-diazatricyclo[6.5.0.0^1^,^4^]tridecane

Pale yellow semisolid: 60% yield; ^1^H NMR (D_2_O, 600 MHz): δ 0.68 (t, 3H), 1.15 (m, 18 H), 1.38-1.71 (m, 8H), 1.92 (t, 1H), 2.45 (d, 1H), 2.62 (t, 2H), 2.70 (d, 1H), 3.31-3.46 (m, 5H), 3.60 (t, 1H); ^13^C NMR (D_2_O, 600MHz): δ 14.3, 20.1, 22.8, 28.8, 29.4, 30.0, 32.1, 36.5, 45.5, 47.2, 49.1, 53.3, 54.9 ppm; ESI-MS (m/z): 320 M+:Anal.Calcd for C_21_H_40_N_2_: C 78.68, H 12.58, N 8.74; found: C 78.60, H 12.63, N 8.82.

**4:**n=11. (RC_14_): 2-dodecyl-4,8-diazatricyclo[6.5.0.0^1^,^4^]tridecane

Pale yellow semisolid: 61% yield; ^1^H NMR (D_2_O, 600 MHz): δ 0.71 (t, 3H), 1.16 (m, 22 H), 1.39-1.70 (m, 8H), 1.93 (t, 1H), 2.41 (d, 1H), 2.60 (t, 2H), 2.70 (d, 1H), 3.30-3.45 (m, 5H), 3.57 (t, 1H); ^13^C NMR (D_2_O, 600MHz): δ 14.1, 20.1, 22.8, 28.8, 29.6, 30.1, 32.2, 36.6, 45.4, 47.3, 49.0, 53.5, 54.7 ppm; ESI-MS (m/z): 348 M+:Anal.Calcd for C_23_H_44_N_2_: C 79.24, H 12.72, N 8.04; found: C 79.12, H 12.67, N 7.98.

**5:**n=13. (RC_16_): 2-tetradecyl-4,8-diazatricyclo[6.5.0.0^1^,^4^]tridecane

Pale yellow semisolid: 65% yield; ^1^H NMR (D_2_O, 600 MHz): δ 0.75 (t, 3H), 1.17 (m, 26 H), 1.41-1.73 (m, 8H), 1.92 (t, 1H), 2.40 (d, 1H), 2.58 (t, 2H), 2.72 (d, 1H), 3.29-3.46 (m, 5H), 3.56 (t, 1H); ^13^C NMR (D_2_O, 600MHz): δ 14.4, 20.2, 22.6, 28.9, 29.4, 30.0, 32.1, 36.5, 45.5, 47.2, 49.1, 53.6, 54.9 ppm; ESI-MS (m/z): 377 M+:Anal.Calcd for C_25_H_48_N_2_: C 79.72, H 12.84, N 7.44; found: C 78.15, H 12.10, N 7.62.

**6:**n=15. (RC_18_): 2-hexadecyl-4,8-diazatricyclo[6.5.0.0^1^,^4^]tridecane

Pale yellow semisolid: 67% yield; ^1^H NMR (D_2_O, 600 MHz): δ 0.74 (t, 3H), 1.15 (m, 30 H), 1.41-1.72 (m, 8H), 1.93 (t, 1H), 2.59 (d, 1H), 2.62 (t, 2H), 2.70 (d, 1H), 3.30-3.46 (m, 5H), 3.58 (t, 1H); ^13^C NMR (D_2_O, 600MHz): δ 14.0, 20.0, 22.8, 28.8, 29.5, 30.1, 32.1, 36.5, 45.5, 47.3, 49.2, 53.5, 54.7 ppm; ESI-MS (m/z): 404 M+:Anal.Calcd for C_27_H_52_N_2_: C 80.13, H 12.95, N 6.92; found: C 80.15, H 12.99, N 6.89.

**7:**n=17. (RC_20_): 2-octadecyl-4,8-diazatricyclo[6.5.0.0^1^,^4^]tridecane

Pale yellow semisolid: 64% yield; ^1^H NMR (D_2_O, 600 MHz): δ 0.78 (t, 3H), 1.20 (m, 34 H), 1.40-1.73 (m, 8H), 1.92 (t, 1H), 2.40 (d, 1H), 2.61 (t, 2H), 2.71 (d, 1H), 3.29-3.47 (m, 5H), 3.55 (t, 1H); ^13^C NMR (D_2_O, 600MHz): δ 14.1, 20.0, 22.7, 28.8, 29.7, 30.0, 32.1, 36.6, 45.4, 47.4, 49.1, 53.6, 54.6 ppm; ESI-MS (m/z): 432 M+:Anal.Calcd for C_29_H_56_N_2_: C 80.48, H 13.04, N 6.47; found: C 80.45, H 13.12, N 6.59.

The results of the characterization analysis on each compound confirmed the structures reported in Figure 1.
